# Emerging Roles of TRIM56 in Antiviral Innate Immunity

**DOI:** 10.3390/v17010072

**Published:** 2025-01-07

**Authors:** Dang Wang, Kui Li

**Affiliations:** Department of Microbiology, Immunology and Biochemistry, University of Tennessee Health Science Center, Memphis, TN 38163, USA

**Keywords:** TRIM56, restriction factor, virus, TLR3, TRIF, cGAS, STING

## Abstract

The tripartite-motif protein 56 (TRIM56) is a RING-type E3 ubiquitin ligase whose functions were recently beginning to be unveiled. While the physiological role(s) of TRIM56 remains unclear, emerging evidence suggests this protein participates in host innate defense mechanisms that guard against viral infections. Interestingly, TRIM56 has been shown to pose a barrier to viruses of distinct families by utilizing its different domains. Apart from exerting direct, restrictive effects on viral propagation, TRIM56 is implicated in regulating innate immune signaling pathways that orchestrate type I interferon response or autophagy, through which it indirectly impacts viral fitness. Remarkably, depending on viral infection settings, TRIM56 either operates in a canonical, E3 ligase-dependent fashion or adopts an enzymatically independent, non-canonical mechanism to bolster innate immune signaling. Moreover, the recent revelation that TRIM56 is an RNA-binding protein sheds new light on its antiviral mechanisms against RNA viruses. This review summarizes recent advances in the emerging roles of TRIM56 in innate antiviral immunity. We focus on its direct virus-restricting effects and its influence on innate immune signaling through two critical pathways: the endolysosome-initiated, double-stranded RNA-sensing TLR3-TRIF pathway and the cytosolic DNA-sensing, cGAS-STING pathway. We discuss the underpinning mechanisms of action and the questions that remain. Further studies understanding the complexity of TRIM56 involvement in innate immunity will add to critical knowledge that could be leveraged for developing antiviral therapeutics.

## 1. Introduction

During their co-evolution with diverse microorganisms, vertebrate hosts have acquired exquisite mechanisms that fend off infections, including those by viruses. Almost immediately after their incursion into susceptible hosts, viruses are met with a barrage of protective responses, which are launched by the host to tackle the invaders. This intrinsic, frontline defense, termed innate immunity, mediates antigen-nonspecific recognition of and response to foreign insults and involves the engagement, activation, and action of specific host cell types, proteins, and intracellular signaling pathways [1,2]. Of the numerous, complex innate immune mechanisms of mammalian cells that resist viral infections, the rapid induction of antiviral cytokines such as the type I and type III interferons (IFNs) is most prominent and important. This process is initiated by diverse families of pattern recognition receptors (PRRs) including, but not limited to, the retinoic acid-inducible gene-I (RIG-I)-like receptors (RLRs), Toll-like receptors (TLRs), and cytosolic DNA sensors (CDSs) [3,4,5,6]. These PRRs detect distinct viral pathogen-associated molecular patterns (PAMPs) bearing specific structural characteristics, such as double-stranded (ds) and single-stranded viral RNAs and viral DNAs. Subsequently, they recruit their cognate adaptors, including mitochondrial antiviral signaling protein (MAVS, also known as IPS-1/VISA/Cardif) for the RLRs, Toll/interleukin-1 receptor (TIR) domain-containing adaptor protein inducing interferon-β (TRIF, also known as TICAM1) for TLR3 and TLR4, myeloid differentiation primary response protein 88 (MyD88) for TLRs other than TLR3, and stimulator of interferon genes protein (STING, also known as TMEM173/MPYS/MITA/ERIS) for cyclic GMP-AMP synthase (cGAS), a major CDS, respectively [7,8,9]. Downstream, through processes facilitated by other critical adaptors and scaffolding proteins, TANK-binding kinase 1 (TBK1) and inhibitors of κB kinase (IKK)-related kinases, such as IKKα, IKKβ, IKKε, and IKKγ, are activated. These lead to phosphorylation and activation of various latent transcription factors, including the IFN-regulatory factors (IRFs) and nuclear factor-κB (NF-κB) [10]. These transcription factors then translocate into the nucleus and play essential roles in the transcription of type I and type III IFNs and inflammatory cytokines/chemokines [2,11].

Many of the innate immune signaling events that connect sensors to the IRFs and NF-κB transcription factors are governed by the activities of kinases and ubiquitin-modifying enzymes, which regulate the activation status of key intermediates in signaling cascades [12]. The ubiquitin (Ub) chain that attaches to signaling molecules during activation of each pathway is controlled by a three-step, sequential reaction that depends on the actions of ubiquitin-activating enzyme (E1), ubiquitin-conjugating enzymes (E2s) and ubiquitin ligases (E3s) [13]. The formation of a polyubiquitin chain entails an isopeptide bond between the carboxyl-terminal Glycine residue of one ubiquitin molecule and an internal Lysine residue (Lys or K) or amino-terminal methionine (M1) of another ubiquitin. In addition to M1, the existence of seven Ks in ubiquitin allows for a variety of ubiquitin linkages, resulting in K6-, K11-, K27-, K29-, K33-, K48-, and K63-linked ubiquitin chains [14]. The substrate specificity of ubiquitination is mostly determined by the E3 ligase involved, which recognizes protein substrates and assists or directly catalyzes the transfer of ubiquitin from an E2 to the substrate [15,16]. Of the ~600 predicted E3 ligases, the really interesting new gene (RING) type ubiquitin ligases are most represented and categorized by the RING finger catalytic domain they operate on. One family of the RING-type ubiquitin ligases is comprised of the tripartite-motif proteins (TRIMs), which include >70 members and are characterized by a highly conserved N-terminal RBCC (RING, B-box, and coiled-coil) motif [17,18]. Given their functions as E3 ligases, understandably, a subset of TRIMs have been implicated in regulating the activity of key signaling molecules in antiviral innate immunity [19,20,21,22]. For example, TRIM65-mediated K63-linked ubiquitination of melanoma differentiation-associated gene 5 (MDA5), an RLR member, is a key step for the initiation of intracellular antiviral response to some RNA viruses [23]; on the other hand, TRIM40 attenuates RLRs signaling by promoting K48- and K27-linked ubiquitination and degradation of RIG-I and MDA5 [24]. Notably, the study of TRIM proteins in host immune defense is one of the fastest-growing subjects in the field of antiviral immunity [25,26,27]. Studies in recent years have demonstrated that TRIM56, an E3 ligase capable of direct RNA binding [28], also plays important parts in host innate antiviral defense through catalytically-dependent and -independent mechanisms. Besides acting as a restriction factor that directly puts a check on a number of RNA viruses [28,29,30,31,32,33], TRIM56 also heightens the induction of IFN antiviral responses via the cGAS-STING [34,35,36,37] and TLR3-TRIF [35,37,38] pathways and regulates autophagy [39,40], in ways that indirectly control viral fitness. In this review, we discuss recent advances in deciphering the biological functions and antiviral actions of TRIM56 in innate immunity against viral infections. A systematic and comprehensive understanding of the roles of TRIM56 in multi-layered host defense will help elucidate the antiviral mechanisms of this TRIM protein and provide clues that potentially inform the development of new antiviral therapeutics.

## 2. Structure and Expression of TRIM56

**Figure 1:**
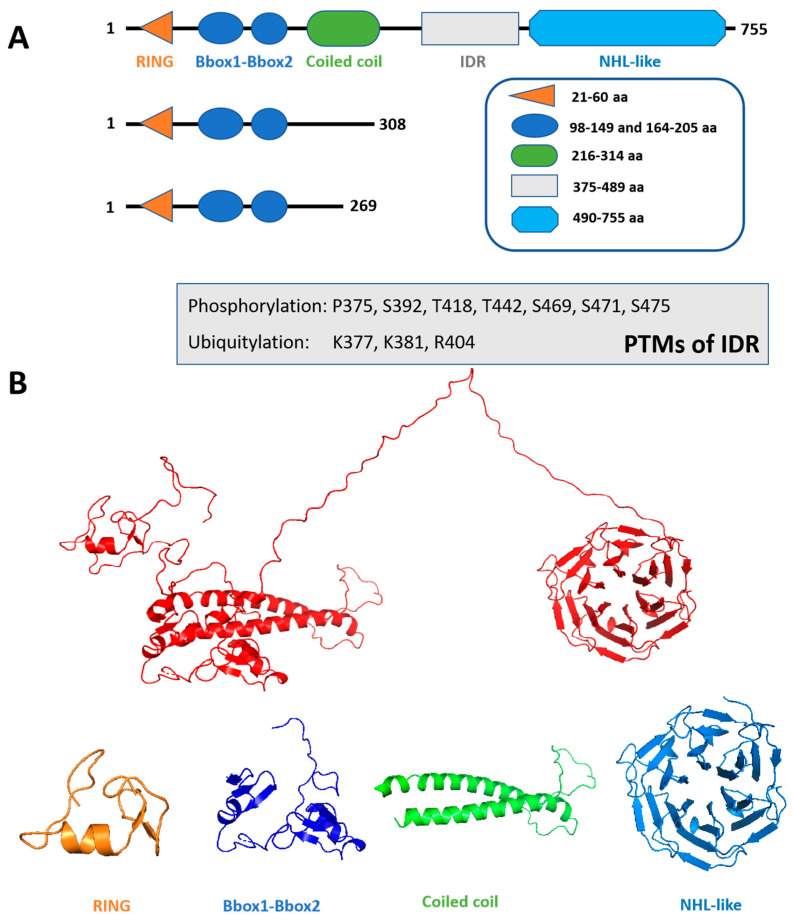
Schematic representation of the structural aspects of human TRIM56 protein. (**A**) Human TRIM56 contains a really interesting new gene (RING) domain, two B-box domains (B-Box 1 and B-box 2), and a coiled-coil domain (CCD) in its N-terminal half, while its C-terminal half encompasses an intrinsically disordered region (IDR) and an NHL repeats (NHL)-like domain. The putative IDR of TRIM56 was predicted by DISOPRED3. Also, in (**A**), the domain architectures of two additional isoforms of human TRIM56 devoid of the C-terminal half (https://www.uniprot.org/uniprot/Q9BRZ2 (accessed on 9 December 2024)) are shown below the 755-aa long reference sequence. (**B**) The tertiary structure of TRIM56 was constructed by template-based tertiary structure modeling using RaptorX servers (http://raptorx.uchicago.edu/ (accessed on 9 December 2024)).

As with a majority of other TRIM proteins, TRIM56 harbors the evolutionarily conserved tripartite structural domains in its N-terminal region, composed of a RING finger, two B-boxes, and a coiled-coil domain (CCD) that are collectively known as the RBCC or TRIM domains (Figure 1A) [18,19]. The RING domain is characterized by a conserved pattern of histidine and cysteine residues, which coordinates the two zinc ions in a cross-supported manner and confers E3 ligase activity [15]. The physiologic substrate(s) for the TRIM56 E3 ligase remains unclear; several proteins in host innate immune signaling have been reported to be modified by this ubiquitin ligase, including STING [36], cGAS [34], and TRIM56 itself [31]. The two B-box domains of TRIM56 are zinc fingers similar to the RING domain and have tandem B-box1–B-box2 arrangements, whereas many TRIM proteins only possess B-box type 2 (B-box2) [19,41]. Thus far, no biological functions have been ascribed to the B-box domains of TRIM56. TRIM56 contains a central CCD that is suggested to enable TRIM protein multimerization necessary for biological function [42,43]. Notably, the CCD is indispensable for TRIM56-mediated positive regulation of innate antiviral immune response downstream of the TLR3 pathway (see below) [38].

In contrast to the structurally conserved N-terminal half, the C-terminal portion of TRIM proteins exhibits high variability in domain architecture, based on which they are divided into up to 11 subfamilies [19]. Among these, the subfamily C-VII members, represented by TRIM32 and TRIM71, harbor NHL (NCL-1, HT2A, and LIN-41) repeats that fold into a six-bladed beta-propeller and afford the capacity to interact with RNAs and other proteins [44]. TRIM56 was initially classified in subfamily C-V due to the lack of a well-characterized domain in its C-terminal region [19]; however, recent data support that an NHL-like domain occupies the bulk of this portion of the protein (Figure 1B) and mediates RNA binding that is critical for the antiviral restriction of some viruses [28,29,45,46,47]. In addition, the NHL-like domain is also involved in TRIM56 interactions with host and viral proteins, as exemplified by the associations of TRIM56 with the TLR3 adaptor TRIF [35] and with the N-terminal protease (Npro) of bovine viral diarrhea virus (BVDV) [31], a member of the pestivirus genus in the Flaviviridae family of positive-stranded, enveloped RNA viruses. Interestingly, structure prediction indicates that an intrinsically disordered region (IDR) exists between the NHL-like and CCD of TRIM56 (Figure 1A), which harbors a significant number of residues subject to post-translational modifications (PTMs) [48,49]. Other than the recent revelation that phosphorylations on two Serine residues in the IDR—Ser-471 and Ser-475—are critical for TRIM56-mediated augmentation of TLR3 signaling [38], the impact of PTMs on TRIM56’s structural conformation, its interactions with cellular and viral factors, and its fundamental biological function remains largely unexplored.

Many TRIM genes encode shorter isoforms that lack one or more exons compared with the canonical sequence [50,51]. Three different isoforms for human TRIM56 as a result of alternative splicing are compiled in the UniProt consortium [52,53] (Figure 1A). The expression and functions of the two shorter isoforms of TRIM56 have yet to be determined. Since both are devoid of the C-terminal IDR and NHL-like domains, they are predicted to lose the RNA binding capability and to have altered protein interaction networks compared with the 755 amino acids (aa) long reference protein.

TRIM56 is broadly expressed in all human tissues examined to date, with the highest protein levels observed in the lungs, stomach, spleen, and ovary, among others [31,54]. Consistent with this, TRIM56 is basally expressed in various human cell lines of diverse tissue origins, e.g., HeLa, Huh7, U2OS, HEK293, THP-1, SVGA, A549, etc., although its expression levels differ [28,31,35,54]. The abundance of TRIM56 is modestly upregulated by viral infections and a variety of stimuli mimicking viral infection settings, including the dsRNA surrogate poly(I:C) and type I IFNs, indicating that TRIM56 is an IFN-stimulated gene (ISG), albeit only moderately IFN-inducible [28,31,35]. In addition, in overexpression settings, TRIM56 localizes in the cytoplasm [31], but a fraction of the protein enters the nucleus post-infection by influenza virus [29] or hepatitis B virus (HBV) [55], a process suggested to be critical for antiviral restriction of the two distinct viruses. Further studies are needed to delineate the exact subcellular localization of endogenous TRIM56 protein and any alterations to it during different viral infections and in various disease settings to aid in understanding its biological functions and regulatory mechanisms.

## 3. TRIM56 Modulates TLR3 Signaling but Not RLR Signaling

**Figure 2:**
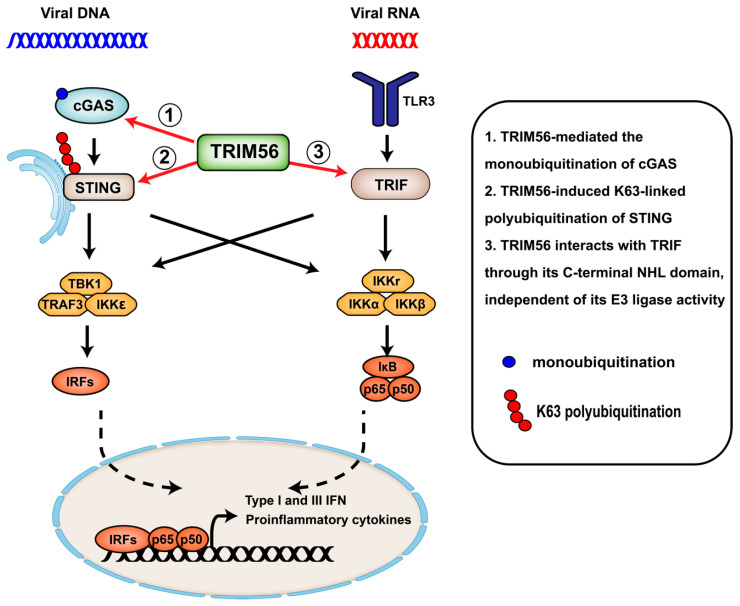
Positive regulation of cGAS-STING- and TLR3-TRIF-dependent innate immune signaling by TRIM56. In the dsRNA-sensing TLR3 pathway, TRIM56 forms a complex with TRIF through its C-terminal NHL-like domain and depends on its coiled-coil domain-mediated scaffolding to promote downstream activation of IRF3 and NF-κB in an E3 ligase-independent manner. In the cytosolic DNA-sensing pathway, TRIM56 interacts with and targets cGAS for monoubiquitination, which initiates its dimerization and cGAMP production. TRIM56 also associates with and promotes K63-linked polyubiquitination of STING, which facilitates STING dimerization, TBK1 recruitment, and subsequent activation of IRF3 and NF-κB.

As the first identified nucleic acid-sensing TLR [56], TLR3 is expressed in the endosomes and/or lysosomes in most cell types and also on the plasma membrane of fibroblasts and some epithelial cell types [2,57]. This PRR recognizes viral dsRNA, either as a part of the viral genome or as viral RNA replicative intermediates produced during the viral life cycle, and modulates innate immune responses to a wide spectrum of RNA viruses as well as some DNA viruses. TLR3 contains an N-terminal extracellular domain (ECD), which is responsible for the binding of dsRNA, as well as a C-terminal cytoplasmic TIR domain essential for downstream signaling. Upon engagement with dsRNA, TLR3 undergoes conformational changes and recruits its adaptor TRIF. The latter, in turn, coalesces signaling complexes together with TNF receptor-associated factor 6 (TRAF6), receptor-interacting serine/threonine-protein kinase 1 (RIPK1), TRAF3, and IKK-related kinases IKKα/β/γ/ε and TBK1, leading to activation of IRF3/IRF7 and NF-κB [2]. Several TRIM proteins have emerged as important regulators of TLR3-dependent antiviral immune response. For example, TLR3 signaling is negatively regulated by TRIM38- and TRIM32-mediated degradation of TRIF via the proteasome and autophagy, respectively [58,59]. On the flip side, studies conducted by our research group have revealed that TRIM56 is a positive regulator of the TLR3 signaling pathway (Figure 2) [35]. Ectopic expression of TRIM56 significantly enhances the expression of IFNs and ISGs following stimulation by extracellular dsRNA, while TRIM56 knockdown or knockout has the opposite effects [35,37,38]. Importantly, TRIM56 depletion greatly compromises the establishment of an antiviral state by the TLR3 ligand and severely impairs chemokine induction via the TLR3 pathway in the context of hepatitis C virus (HCV) infection [35]. Mouse TRIM56, which shares 81% aa identity with human TRIM56 but is 21-aa shorter than the latter, also promotes TLR3 signaling [38]. Of note, the induction of IFN antiviral responses by Sendai virus, a murine respirovirus of the family Paramyxoviridae, or by cytoplasmic delivery of dsRNA, is not affected by manipulation of TRIM56 abundance, indicating that TRIM56 is dispensable for antiviral signaling via the RLRs, RIG-I, and MDA5 [31,35].

Interestingly, mutational analyses of TRIM56 have revealed a catalytically independent, non-canonical mechanism underpins its function in augmenting TLR3 signaling, as TRIM56 mutants bearing Alanine mutations at the two zinc-coordinating Cysteine residues, Cys-21 and Cys-24, in the RING or lacking the entire RING domain exhibit no loss in activity [35]. This is in sharp contrast to the effect of TRIM56 on the cGAS-STING pathway (see the next section), which critically relies on its E3 ligase activity that promotes the ubiquitination of signaling molecules (the so-called “canonical” mechanism). Instead, biochemical analyses suggest that the ability to promote TLR3 signaling depends on the physical interaction between TRIM56 and TRIF via the C-terminal NHL-like domain of TRIM56, the integrity of which is critical [35,38]. This interaction heightens the activation of both IRF3 and NF-κB [35,38], consistent with that the TLR3 pathway bifurcates at TRIF, whereupon TRIM56 acts, to the two downstream signaling arms. Additional requirements for TRIM56 to function in augmenting TLR3-TRIF-dependent antiviral responses include an intact CCD and phosphorylations on two conserved Serine residues in the IDR, Ser-471, and Ser-475, which reside in a segment close to the beginning of the C-terminal NHL-like domain [38]. Exactly how these molecular determinants contribute to the TRIM56 regulation of the TLR3-TRIF axis awaits further investigations, as does the question of what kinase(s) catalyzes these specific phosphorylations on TRIM56. Since the CCD deletion mutant of TRIM56 remains capable of interacting with TRIF despite being abrogated for TLR3 signaling enhancement, prior oligomerization of TRIM56 afforded by the CCD is not a prerequisite for its association with TRIF. Instead, the most plausible scenario is that TRIM56 CCD-mediated scaffolding facilitates the recruitment of additional signaling proteins to TRIF for multi-protein complex assembly, thereby strengthening signaling cascades downstream of this pathway. With regards to the TRIM56 Ser-471 and Ser-475 phosphorylations, data show that both events occur concurrently with bi-phasic kinetics, with the early phase taking place at 0.5–1 h following TLR3 engagement and preceding the TRIM56-TRIF interaction and IRF3 phosphorylation [38]. The precise biological consequence of these PTMs on TRIM56 remains to be elucidated, be it through regulating TRIM56 structural dynamics, altering TRIM56 subcellular movement, or affecting the efficiency of TRIM56, forming a complex with TRIF and/or other signaling proteins.

## 4. TRIM56 Regulates cGAS-STING Signaling

In recent years, multiple DNA sensors have been reported to recognize viral DNA. Of these, the most prominent is the cyclic GMP-AMP synthase (cGAS) [60], whose important role in sensing DNA viruses has been corroborated in numerous studies. After engagement with viral DNA or cellular DNA leaked to the cytoplasm, cGAS catalyzes the production of cyclic GMP-AMP (cGAMP). This second messenger binds to the stimulator of IFN genes (STING), which subsequently recruits TBK1 to phosphorylate and activate IRF3. In addition, STING also associates with the IKK complex and NF-κB-inducing kinase (NIK) to activate NF-κB [61]. Recent data suggest that the activity of cGAS and STING is regulated by PTMs, such as ubiquitination, and several TRIM proteins have been implicated in controlling cGAS-STING-dependent antiviral signaling via this PTM [22]. For example, the mouse-specific TRIM30α binds to STING for K48-linked polyubiquitination at K275, thereby promoting proteasomal degradation of the latter [62]; on the contrary, TRIM32 polyubiquitinates STING with K63-linked polymeric chain at K20, K150, K224, and K236, and in doing so positively regulates cGAS-STING signaling [63]. Similarly, TRIM56 has also been reported to interact with and facilitate K63-linked polyubiquitination of STING at residue K150 (Figure 2), which spurs STING dimerization as well as its forming a complex with TBK1 [36]. However, others have described that TRIM56 does not interact with STING and is unlikely to be directly responsible for the latter’s ubiquitination under more stringent experimental conditions [64]. In addition, TRIM56-deficient cells mount intact IFN response to stimulation by cGAMP despite having a severely impaired capacity in response to cytosolic DNA stimulation, suggesting TRM56 operates upstream of STING in this pathway [34]. Rather, a central portion of TRIM56 (residues 308–531) binds to the N-terminal regulatory domain of cGAS, and this interaction promotes the monoubiquitination of cGAS at residue K335 by the TRIM56 ubiquitin ligase (Figure 2) [34]. This specific PTM of cGAS is required for its dimerization, DNA binding capacity, and cGAMP production. Notably, the effect of TRIM56 on the cGAS-STING pathway critically relies on its E3 ligase activity, contrasting with its E3 ligase-independent augmentation of TLR3 signaling. This illustrates how TRIM56’s canonical (E3 ligase-dependent) and non-canonical (E3 ligase-independent) mechanisms operate in distinct contexts. By employing different mechanisms, TRIM56 can modulate multiple antiviral pathways, potentially complementing each other to provide a more robust immune response. Elucidating how these mechanisms interact could offer deeper insights into the regulatory roles of TRIM56 in innate immunity. Congruent with the in vitro findings, *Trim56*-deficient mice produce significantly lower levels of IFN-β and exhibit a higher rate of lethality compared to wild-type (WT) animals upon challenge by HSV-1, a dsDNA virus in the family Orthoherpesviridae. In agreement with the in vivo data, bone marrow-derived macrophages (BMDMs) from *Trim56*-deficient mice are defective for IFN-α/β production following HSV-1 mutant γ34.5 virus (mutHSV-1) infection, in contrast to WT BMDMs that allow for significant type I IFN induction [34]. While the discrepancy in the proposed mechanisms by which the catalytic activity of TRIM56 facilitates cGAS-STING signaling needs to be reconciled, knockdown and knockout studies conducted in human cells have corroborated the positive regulatory role of TRIM56 in cytosolic DNA-induced antiviral responses [34,35,36,37]. It is worth noting that in addition to antiviral responses, the cGAS-STING pathway plays significant roles in antibacterial immunity and sterile inflammation caused by mitochondrial DNA release [65,66]. Thus, TRIM56’s regulation of cGAS-STING signaling suggests it may influence these broader immune processes. By modulating this pathway, TRIM56 could help fine-tune immune responses to various pathogens and inflammatory stimuli beyond viral infections.

## 5. Involvement of TRIM56 in Autophagy

Autophagy is an evolutionarily conserved intracellular process that mediates lysosome-dependent degradation of damaged organelles, misfolded proteins, and, in some cases, viral cargo [67]. Besides the diverse homeostatic functions of autophagy, a growing body of evidence suggests that autophagy can have either antiviral or pro-viral capacities in the life cycles of different viruses [68]. Multiple mechanisms have been proposed to explain autophagy-induced antiviral effects, such as facilitating innate immune signaling to enhance antiviral responses and directly degrading viral proteins, both of which promote virus clearance [69]. Several TRIM proteins have been shown to regulate autophagy induced by viral or non-viral stimuli [70,71]. TRIM5α has been suggested to act as a viral cargo receptor and induce autophagic destruction of HIV capsid [72]. TRIM23 promotes p62 (a.k.a., sequestosome 1, SQSTM1)-mediated selective autophagy during viral infection via the TRIM23-TBK1-p62 axis [40]. In a genetic screen, a significant fraction of TRIM proteins can act as a regulator of autophagy; 21 out of 72 TRIM proteins were found to regulate autophagy induced by treatment with pp242, an mTOR inhibitor [72]. Another study identified 24 TRIM proteins to be essential for IFN-γ-induced autophagy in THP1 cells and showed that knockdown of TRIM56 led to borderline but statistically significant reduction in endogenous microtubule-associated protein 1A/1B-light chain 3 (LC3) puncta formation (indicative of autophagy induction) following IFN-γ stimulation [39]. In addition, a third study reported that, out of 61 TRIM proteins tested, overexpression of 31 individual TRIMs in HeLa cells induced green fluorescent protein (GFP)-LC3 puncta formation to variable degrees, as compared to empty vector transfection. Among these, TRIM56 overexpression had an intermediate effect, while knockdown of TRIM56 significantly reduced GFP-LC3 puncta formation during infection by mutHSV-1. By comparison, TRIM56 depletion had a marginal effect on autophagosome formation in the context of influenza A virus (IAV) infection and exhibited no impact following infection by a picornavirus, encephalomyocarditis virus (EMCV) [40]. Taken together, these observations suggest that TRIM56 participates in modulating host autophagic response during some viral infections. Since the influences of autophagy on viral replication are virus-specific and the autophagy-associated regulation of innate immune signaling differs among cell types and viral entry routes, etc., the effects of TRIM56 on autophagy, if any, will need to be clarified in further studies using biologically relevant cell types and animal models for the specific virus concerned. Recently, a link between STING and autophagy has been suggested. In this role, STING functions as an autophagy receptor by interacting with LC3 through its LC3-interacting regions, promoting selective autophagy and mediating its own degradation via autophagy pathways [73]. Given the reported TRIM56-STING connection [36], it is tempting to ask the question of whether the reported effects of TRIM56 on autophagy in some viral infection settings stem from its modulation of STING activity, which links immune signaling and autophagic processes.

## 6. TRIM56 as a Restriction Factor of Viral Infections

Besides the rapid induction of IFNs and, subsequently, ~300 ISGs, the products of which act in concert to impede virus replication and spread, host species have evolved numerous restriction factors that are basally expressed at levels functioning as intracellular blocks to specific viruses at various stages of the viral life cycle. Not surprisingly, a considerable fraction of the virus restriction factors characterized to date can themselves be upregulated by IFNs. However, their basal expression levels are, in general, higher than those of classical ISGs, and their extent of upregulation by IFNs is moderate. Restriction factors have been shown to hinder intracellular virus multiplication by targeting viral products or host cellular factors necessary for the invader to proceed through its life cycle. Herein, we refer to this antiviral barrier function as “direct” virus restriction since it is designed to tackle a specific (group of) virus(es). Notably, this mode of action differs from antiviral effects executed by enhancing host innate antiviral immune signaling and induction of IFN response, which puts a check on viruses “indirectly” and typically in an indiscriminate fashion. TRIM proteins were first recognized for their role as a family of virus restriction factors approximately two decades ago, when TRIM5a was discovered as a species barrier to HIV by disrupting the nucleocapsid uncoating process post-entry [74]. By that time, the vast majority of the ~70 TRIMs had not been characterized for their functions, and knowledge of the viral PRRs and their downstream pathways was at their infant stage. Understandably, early studies on the interplay of TRIM proteins and viral infections during the 2000s had mostly focused on their anti-retroviral activities [19]. Subsequently, as the field heated up, a growing number of TRIM proteins have been shown to play antiviral roles in restricting RNA and DNA viruses beyond retroviruses.

TRIM56 was first characterized as a virus restriction factor when our research group investigated how BVDV, a Flaviviridae member of veterinary importance, blocks the induction of IFN antiviral response by its Npro protein. The latter exerts its immune antagonism by forming a complex with and promotes the polyubiquitination and subsequent proteasomal degradation of IRF3 [75], a key transcription factor controlling the synthesis of type I and type III IFNs. In this study [31], we aimed to search for the cellular E3 ubiquitin ligase(s) usurped by Npro for this process. TRIM56, as a hitherto putative E3 ligase protein, was identified as an interaction partner of Npro. We observed that TRIM56 possesses E3 ligase activity towards its own polyubiquitination but is dispensable for Npro-induced IRF3 loss. We did not find TRIM56 to target Npro for degradation, either. Instead, we uncovered that TRIM56 inhibits BVDV replication by hindering intracellular viral RNA replication. Mutational analyses demonstrated that TRIM56 depends on its RING domain-derived E3 ligase activity as well as the integrity of its C-terminal portion [31], later revealed to house the NHL-like repeats [29] for its inhibitory effect on BVDV. This antiviral barrier property, as shown in cell culture infection models, does not stem from general augmentation of IFN response, a conclusion also supported by the observations that TRIM56 does not restrict vesicular stomatitis virus (VSV, a Rhabdoviridae member) [29,31] or HCV [31]. Subsequent studies delineating the impacts of TRIM56 on viruses related to BVDV have revealed that five mosquito-borne viruses in the orthoflavivirus genus of family Flaviviridae are susceptible to this host restriction factor, including yellow fever virus (YFV), Dengue virus (DENV, of both serotypes 1 and 2), Zika virus (ZIKV, of both African and Asian lineage) [28,30], and most recently, Usutu virus (USUV) and West Nile virus (WNV) [76]. Analyses of the interplay between TRIM56 and additional RNA viruses led to the revelations that TRIM56 also restricts two coronaviruses—human coronavirus OC43 (HCoV-OC43) [30] and porcine epidemic diarrhea virus (PEDV) [33]—two Orthomyxoviridae members—influenza A and B viruses (IAV and IBV) [29] and Sindbis virus (an alphavirus in the family Togaviridae) [45]—but has no demonstrable antiviral effects against human metapneumovirus and murine respirovirus [29], representatives of the family Paramyxoviridae. The impact of TRIM56 on viruses in the family Picornaviridae has also been examined. In an early study, the tetracycline-regulated conditional overexpression of TRIM56 was not found to inhibit EMCV, a prototype member of the genus Cardiovirus, in HEK293 cells [30]. In comparison, a very recent study reported that transient overexpression of TRIM56 in HeLa cells reduces the yields of Coxsackievirus B3, a virus belonging to the Enterovirus genus, by mediating the polyubiquitination and proteasomal degradation of viral 3D RNA-dependent RNA polymerase [32]. It is unclear if the disparate phenotypes observed reflect intrinsic differences in susceptibility to TRIM56 between the two genera of picornaviruses or stem from cell type-specific differences.

Apart from its antiviral activities against RNA viruses, TRIM56 has been reported to inhibit HIV-1 and HBV, effects not attributable to enhancement of cGAS-STING signaling or increased expression of IFNs and/or ISGs [55,77]. Overexpression of TRIM56 inhibits the expression of late (but not early) HIV-1 genes, reducing viral particle production and spread [77]. Endogenous TRIM56 is insufficient for inhibiting HIV-1 replication, but interestingly, appears to be required for optimal anti-HIV activity of exogenous IFN-α in MT4, a T cell leukemia cell line [77]. This latter observation likely is HIV-specific since another study examining HeLa cell lines with complete knockout for TRIM56 showed no significant impairment in the establishment of a general antiviral state by IFN-α, measured against VSV [37]. In the case of HBV inhibition, TRIM56 has been suggested to suppress the viral core promoter indirectly by enhancing phosphorylation of the RelA/p65 subunit of NF-κB transcription factor, perhaps by promoting K48-linked polyubiquitination of IκBα via its RING-derived E3 ligase activity [55]. Currently, it is unclear to what extent this proposed NF-κB regulation may operate in other virus infection settings, as TRIM56 has no demonstrable effect on IκBα phosphorylation [55], the canonical mechanism underlying NF-κB activation. Consistent with the latter observation, TRIM56 overexpression does not alter the extent of NF-κB activation following stimulation by inflammatory cytokines, IL-1α, or TNF-α [38].

Characterizing the TRIM56 determinants essential for each of its antiviral activities helps unscramble the underpinning biology. Domain mapping of TRIM56 has been performed regarding its interplay with BVDV and related flaviviruses (YFV, DENV, and ZIKV), HCoV-OC43, IAV and IBV, and HBV [28,29,30,31,55]. These analyses suggest that TRIM56 has evolved exquisite domains/motifs that it employs to impact various viral (and/or host) processes that dictate pathogen fitness (Table 1). Not surprisingly, TRIM56-mediated restriction of similar viruses depends on shared domains and/or activities. For instance, TRIM56 requires its RING domain and associated E3 ligase activity as well as the integrity of the C-terminal NHL-like domain to target the viral RNA replication stage of Flaviviridae members for inhibition, which also necessitates a central portion of the protein (residues ~355–433) that overlaps with the IDR based on analyses of the effects of TRIM56 mutants on YFV and DENV [30]. By comparison, the RING domain (and associated E3 ligase activity) alone of TRIM56 is sufficient for implementing the full-length protein’s antiviral function against HCoV-OC43, which impedes a late step in the viral life cycle post viral RNA replication [30]. In contrast, a C-terminal tail fragment of as few as ~63 aa, but not any other portions of TRIM56, is required for hampering IAV and IBV RNA synthesis in the nucleus [29]. Given that the CCD is dispensable for the antiviral activities against all above-mentioned RNA viruses, it is plausible that TRIM56 does not need to dimerize or multimerize to fulfill its “direct” antiviral actions (which is in stark contrast to its positive regulation of TLR3-TRIF immune signaling). However, the substrate(s) for the TRIM56 E3 ligase responsible for the restriction of flaviviruses and HCoV-OC43 remains elusive. The C-terminal NHL-like repeats are capable of directly binding ZIKV RNA, and this property is important for TRIM56’s inhibitory action on flavivirus RNA replication [28]. Whether TRIM56 also binds to the RNAs of related viruses to impose viral restriction and whether there is a conserved sequence motif on viral RNAs that TRIM56 recognizes will require further study. Along this line, it is worth mentioning that overexpression of TRIM56 has been shown to reduce Sindbis virus fitness, an effect suggested to stem from the ability of TRIM56 to bind genomic RNA of this alphavirus [45]. Taken together, the RNA-binding activity of the NHL-like domain is an important component of TRIM56’s antiviral restriction of a significant number of RNA viruses. Outstanding are the questions as to why the structural integrity of the entire NHL is critical for hindering the RNA synthesis of some (e.g., Flaviviridae members) but not other (e.g., IAV and IBV) viruses and whether RNA-binding may, in some way, modulate the catalytic activity of TRIM56 ubiquitin ligase, as is the case suggested for TRIM25 [78]. It is noteworthy that while several NHL-containing TRIM proteins partake in regulating the miRNA pathway, the restriction of ZIKV by TRIM56 is not compromised in endoribonuclease DICER1-knockout cells, suggesting TRIM56’s anti-flavivirus action operates independent of the biogenesis of or cellular gene expression regulation by miRNAs [28].

In light of its broad activities against multiple viral families, TRIM56 presents a promising node for potential therapeutic applications. Enhancing TRIM56 expression or mimicking its action could lead to novel antiviral strategies, particularly against emerging viral diseases for which existing therapies are insufficient. For example, TRIM56-based interventions might offer new treatments for infections by influenza and coronaviruses, including COVID-19. Still, a deeper understanding of the precise antiviral mechanisms of TRIM56, including but not limited to elucidating its cellular and/or viral targets, is needed to fully realize its therapeutic potential and avoid unwanted proinflammatory side effects.

## 7. Viral Targeting of TRIM56

As the first viral protein identified to interact with TRIM56, BVDV Npro promotes proteasome-dependent degradation of TRIM56. In HeLa cells ectopically expressing Npro, the abundance of endogenous TRIM56 protein was profoundly reduced, a phenotype that could be reversed by treatment with epoxomicin, a potent proteasome inhibitor [31]. This revelation adds to our understanding of why all pestiviruses have evolved to encode this small viral protein, which is not part of the viral replicase complex and not essential for viral RNA replication. Aside from its well-established role in viral polyprotein processing by catalyzing cis-cleavage at the Npro-Capsid junction (note that the protease activity of Npro has not been shown to function *in trans*), Npro is now known to operate as a multifunctional immune antagonist that disarms not only IRF3-dependent, overall IFN antiviral responses but also a specific host defense mechanism, i.e., TRIM56-mediated restriction of viral RNA replication, by co-opting the proteasome degradation pathway of host species. Conceivably, this two-pronged viral immune evasion strategy affords pestiviruses a maximal survival advantage. Very recently, the CVB3 virus has been suggested to induce TRIM56 cleavage in HeLa cells late post-infection. Overexpression of the viral 3C protease, but not a catalytically inactive mutant, alone was sufficient to recapitulate the TRIM56 scission [32], illustrating that 3C protease-mediated cleavage of TRIM56 is a picornaviral tactic to overcome this specific antiviral protection barrier. In light of the multifaceted involvement of TRIM56 in virus-host interactions, it is anticipated that additional viruses will be found to counteract this versatile antiviral protein, perhaps in different ways, for their unabated multiplication and survival.

## 8. Conclusions and Perspectives

It has become increasingly clear that TRIM56 is an important, multifunctional player in host innate immune responses fending off viral infections. Whereas the last ~one and a half decades have witnessed a number of exciting discoveries of the various new roles that TRIM56 plays in antiviral immune signaling and as a direct virus restriction factor, much remains to be learned about the detailed, regulatory and effector mechanisms by which TRIM56 operates in these processes. Precisely how TRIM56 is activated and enlisted and acts to combat a broad range of viruses remains murky. The substrates for the E3 ligase of TRIM56 that underlie the “direct” antiviral effects are, by and large, elusive. The discovery of TRIM56’s direct viral RNA binding ability provides new insights into its antiviral mechanism against some RNA viruses, and further studies are necessary to determine whether RNA binding by the NHL-like domain of TRIM56 modulates its E3 ligase activity. To fully understand the functions and regulation of TRIM56, it will be helpful to study the different isoforms of TRIM56. Specifically, whether the two shorter isoforms exhibit tissue- and/or cell-type-specific distribution and how their expression and activity are regulated following various viral and/or immune stimuli. Also, clarifications on the contradictory roles reported for the TRIM56 ubiquitin ligase in cGAS-STING signaling are much needed, as is the exact biology underpinning the non-canonical, E3 ligase-independent involvement of TRIM56 in the TLR3-TRIF pathway. Last but not least, investigating the contributions of TRIM56 to regulating viral fitness and immune responses in appropriate animal models will help establish the biological relevance of many of the findings suggested in cell culture models and whether specific aspects of TRIM56-virus interplay hold the potential for therapeutic targeting.

## Figures and Tables

**Table 1:** Antiviral activities of TRIM56 against various viruses.

Study [Ref #]	Virus Species	Virus Classification (Family, Genus)	Host Cell (or Animal)	Proposed Mechanism	Domain(s) of TRIM56 Required for Antiviral Activity
Wang et al. 2011 [31]	Bovine viral diarrhea virus	*Flaviviridae*, *pestivirus*	MDBK	Inhibits replication of viral RNA	RING (and E3 ligase activity) and NHL-like domains
Liu et al. 2014 [30]	Yellow fever virus	*Flaviviridae*, *orthoflavivirus*	HEK293, HeLa	Inhibits replication of viral RNA	RING (and E3 ligase activity), IDR, and NHL-like domains
Liu et al. 2014 [30]	Dengue virus type 2	*Flaviviridae*, *orthoflavivirus*	HEK293, HeLa	Inhibits replication of viral RNA	RING (and E3 ligase activity), IDR, and NHL-like domains
Yang et al. 2019 [28]	Dengue virus type 1	*Flaviviridae*, *orthoflavivirus*	HEK293	Inhibits replication of viral RNA	Not determined
Yang et al. 2019 [28]	Zika virus	*Flaviviridae*, *orthoflavivirus*	HEK293, HeLa, 293T, 293T-DICER-KO, SVGA, SK-N-SH	Directly binds to and inhibits replication of viral RNA	RING (and E3 ligase activity) and NHL-like domains
Zoladek et al. 2024 [76]	Usutu virus West Nile virus	*Flaviviridae*, *orthoflavivirus* *Flaviviridae*, *orthoflavivirus*	HEK293T HEK293T	Not determined Not determined	Not determined Not determined
Liu et al. 2014 [30]	Human coronavirus OC43	*Coronaviridae*, *betacoronavirus*	HEK293, HeLa	Inhibits a late stage of viral life cycle post viral RNA synthesis	RING (and E3 ligase activity)
Xu et al. 2022 [33]	Porcine epidemic diarrhea virus	*Coronaviridae*, *alphacoronavirus*	Marc-145	Enhances TLR3-TRAF3-mediated IFN-β antiviral response	RING and C-terminal half
Liu et al. 2016 [29]	Influenza A virus and Influenza B virus	*Orthomyxoviridae*, *alphainfluenzavirus* *Orthomyxoviridae*, *betainfluenzavirus*	HEK293, HeLa, MDBK	Reduces viral RNA synthesis	C-terminal tail of ~63 aa alone
Garcia-Moreno et al. 2019 [45]	Sindbis virus	*Togaviridae*, *alphavirus*	HeLa	Binds to viral RNA	Not determined
Wang et al. 2024 [32]	Coxsackievirus B3	*Picornaviridae*, *enterovirus*	HeLa	Promotes ubiquitination and proteasomal degradation of viral 3D polymerase	Not determined
Kane et al. 2016 [77]	Human immunodeficiency virus type 1	*Retroviridae*, *lentivirus*	HEK293T, GHOSTX4	Inhibits the expression of late HIV-1 genes	Not determined
Seo et al. 2018 [34]	Herpes simplex virus-1	*Herpesviridae*, *simplexvirus*	BMDMs, mice	Monoubiquitinates cGAS to promote type I IFN response	Not determined
Tian et al. 2022 [55]	Hepatitis B virus	*Hepadnaviridae*, *orthohepadnavirus*	HepG2-NTCP, primary human hepatocytes	Inhibits HBV core promoter by ubiquitinating IκBα and activating NF-κB p65 subunit	RING (and E3 ligase activity) and C-terminal tail region
